# The Dreamwork of the Symptom: Reading Structural Racism and Family History in a Drug Addiction

**DOI:** 10.1007/s11013-023-09820-w

**Published:** 2023-04-06

**Authors:** Jesse Proudfoot

**Affiliations:** Department of Sociology, Durham University, 32 Old Elvet, Durham, DH1 3HN UK

**Keywords:** Addiction, Structural violence, Racism, Psychoanalysis, Illness narratives

## Abstract

A key tenet of critical health research is that individual symptoms must be considered in light of the social and political contexts that shape or, in some cases, produce them. Precisely how oppressive social forces give rise to individual symptoms, however, remains challenging to theorize. This article contributes to debates over the interpretation of symptoms through a close reading of the case of Leon, an African American man struggling with an addiction to crack cocaine. Leon presented a complex illness narrative in which his addiction was clearly a product of structural racism, but also the result of dynamics within his family. Drawing on critical reevaluations of Freud’s concept of the dreamwork, I call attention to the surface elements of Leon’s narrative—what I term the surface of the symptom—and to the formal mechanisms by which latent contents (such as the social, the political, and the personal) are transformed into the manifest form of his symptom. This formal mode of reading offers a productive way of approaching questions of demystification and interpretation, one that holds in tension the register of social causation with the singularities of individuals and their symptoms.

## Introduction: The Surface of the symptom

Anger management—I don’t agree with! We should—I should be angry! I should be angry at a lot of things that’s happened to me! We as Black men—we’re angry... We’re angry at how society… has allowed the drugs to flourish through our neighborhoods—has allowed the job rate to deplete—has allowed the resources in our neighborhoods to not be there… They say, ‘anger management’, but they never address why we’re angry… There’s a reason why we’re angry!

Leon^1^ had long been critical of the substance use treatment program that he was required to attend, and the Anger Management course captured its contradictions perfectly: Why should poor African American men like him, living in devastated communities, exposed to all the violence of the drug war, frequently cycling in and out of the prison system, not be angry? Lifting his pant leg to reveal his electronic ankle monitor, he exclaimed, “This is my slave bracelet! …The 13th Amendment says they gave us freedom; it says, ‘Slavery is abolished’—unless you in jail!” Leon, an African American man in his mid-fifties, had just been released from prison for minor theft, a recurring event in his life that stemmed from his addiction to crack cocaine. His experiences of prison and addiction had given rise to a political analysis that wove together critiques of structural racism and neoliberalism to make sense of mass incarceration, the organized abandonment of African American neighborhoods by the state, and his own relationship to drugs. To Leon, Black neighborhoods were intentionally saturated with drugs, in order to render communities dependent, passive and legally precarious: “The drugs control the people. Police control the gangs. Who control the police? Those with the money. Political power.”

In his own way, then, Leon had accomplished what critical scholars of health have long advocated: an analysis of the social relations that lie behind illness. Rather than viewing his addiction as an ahistorical, biological, personal failing, he had submitted his addiction to a form of critique that uncovered the social forces that gave rise to it, reaching similar conclusions as many critical addictions researchers (Alexander 2010; Bourgois 1995; Bourgois and Schonberg 2009; Garcia 2010; Raikhel and Garriott 2013; Singer et al. 1992). If his lay analysis was somewhat more blunt than the theorizations provided by academics, it nevertheless identified essential truths about systemic racism and the political economy of substance use in the contemporary United States. And yet, while these explanations provided Leon with a framework for understanding his illness, this insight had not changed his own compulsive relationship to drugs: three months after we began our conversations, Leon relapsed, violated his parole, and returned to prison.

In this article, I attempt to make sense of Leon’s addiction to drugs, situating it, as he did, within the context of structural violence and racism. In doing so, I turn to arguments within medical anthropology about the relationship between symptoms and oppression (Scheper-Hughes 1993; Singer et al. 1992), illnesses and reification (Taussig 1992), and broader questions about the interpretation of illnesses (Kleinman 1988; Good 1994; Biehl 2005). While illnesses are frequently—and, I argue, correctly—treated within this literature as social rather than purely individual phenomena, Leon’s case illustrates the challenges posed by the complex imbrication of the social with the personal in the formation of symptoms. Drawing on critical re-evaluations of Freud’s *dreamwork* and his often-overlooked insistence on the importance of the formal mechanisms by which dreams are produced, I suggest that the dreamwork provides an invaluable method for understanding this relationship—specifically, by directing our attention to what I term the *surfaces* of symptoms and not only what we presume those surfaces conceal. In Leon’s case, this formal analysis allowed me to see how the political narratives that both he and I employed to understand his suffering moved too quickly from subjective experience to macrostructural forces, in the process, missing what Paul Rabinow calls the “particular mediations” (2007:124) that connect them. This approach carries with it, I claim, an invitation to critically revisit the status of the individual and the individual symptom, in keeping with the tradition of ethnographies of a single person (e.g. Biehl 2005; Crapanzano 1980). In struggling to make sense of Leon’s addiction, I came to see that attending to the individual is not necessarily synonymous with mystification; rather, the problem of mystification may be better understood as a matter of which *modes of reading* we bring to the symptom.

## Methods

I met Leon in Chicago in 2012 at a substance use treatment facility for men recently released from prison, where I conducted 18 months of fieldwork on addiction and structural violence, including one-on-one interviews with 21 residents of the facility. Over a period of three months, Leon and I spoke informally on numerous occasions, sat for six interviews, and went on two daytrips in the city to visit significant places from his life. After I left Chicago, we communicated occasionally by letter and telephone, before losing touch as each of us moved houses and as he returned, periodically, to prison.

My interviews at the facility were modeled on psychoanalytic principles of free association. At the outset, I asked each interlocutor simply to tell me whatever they believed was relevant to understand them and their relationships to drugs. With Leon, this led to extensive discussions of his life and childhood as well as conversations about racism, politics, and history. As I am not trained as an analyst, these interviews were not offered as therapy in any sense, though many interlocutors commented that they experienced them as therapeutic. Leon, and many of the other men I interviewed, saw the interviews as an opportunity to share their stories, often with the stated desire that in doing so, they could further understandings of addiction and help others to avoid the mistakes they felt they had made.

A central impetus for this research was to provide a space for people to share their experience and reflect on it in a genuinely non-judgmental and non-instrumental setting. Such spaces stand in stark contrast to the scripted forms of engagement often required by state-mandated treatment programs (Summerson-Carr 2012:163–64). All too often, marginalized people like Leon are caught between two unsatisfactory positions: a ‘hostile’ one in which they are punished or ignored, and a ‘sympathetic’ one in which they are heard, but only narrowly, as ‘types’ or exemplars of social issues. In the latter, the problems that people struggle with are treated as the product of the structural inequalities that they face, without consideration of the complex and sometimes contradictory subjective worlds they bring to them. Even when such analyses are motivated by a commitment to social justice—as they are in much critical medical anthropology—this “representational strategy of *idealizing lives*”, as João Biehl and Amy Moran-Thomas describe it, is “limited in terms of addressing singularity… obscuring the nuanced and volatile textures of interlocutors’ own subjectivities” (2009, 275–76 emphasis mine). A key aim of this paper, then, is to make space for the complexity of marginalized people’s experiences—to assert, in other words, their right to an unconscious.

As a middle-class white man, my decision to write about the lived experience of Leon, a formerly incarcerated African American man who uses drugs, demands consideration of positionality and the politics of representation. In their research on media representations of the opioid epidemic, Hansen and Netherland note how “in stories about suburban or rural white drug use, the etiology of the person’s drug use was often explored, while in accounts of drug use among Blacks and Latinos such explanations… were simply missing” (2016, 673). This article attempts to respond to such disparities in representation, offering a grounded, sympathetic analysis of one African American man’s drug use. While good intentions alone do not resolve the many problems that attend “speaking for others” (Alcoff 1991), I am ultimately persuaded by Olúfẹ́mi O. Táíwò’s recent critique of “deference politics”: the practice on the contemporary Left to ‘center marginalized voices’, ‘listen to the most affected’, and refuse to speak for marginalized groups. The motivations behind such politics may be admirable, Táíwò argues, but they also run the very real risk of promoting “moral cowardice” and displacing the responsibility for speaking onto marginalized others—“more often than not, a hyper-sanitized and thoroughly fictional caricature of them” (Táíwò 2021; see too Schulman 2016). For these reasons, I take seriously Gayatri Spivak’s call for those with the privilege to speak to take the “risk to criticize” (1990:62) and not allow the hazards of speaking for the other to result in a “further silencing of already-marginalised groups” (Griffiths 2018:305).

## Symptoms and the Hermeneutics of Suspicion

Leon was a 55-year-old African American man from the South Side of Chicago. He had been addicted to crack cocaine for almost thirty years and had been incarcerated on multiple occasions for minor thefts related to his drug use. His addiction was chronic but distinctly episodic: for long periods of his life, Leon stopped using drugs, worked (generally as a welder), pursued further education, and raised a family with his wife. Then, for reasons he did not understand, these periods of stability would end when he returned to using crack, binged until he ran out of money, and eventually turned to theft and fraud until he was caught and sent to prison again.

Beyond any clinical diagnosis, I argue that Leon’s addiction to drugs should be understood as a ‘symptom’ in the psychoanalytic sense of the term: it was something that caused him to behave in ways that disturbed him and that he did not understand (Fink 2017:76–77, 199). Leon described his first experience of cocaine as being like an “orgasm” that instantly became “the only thing you want”. From 1980 to 2010, his episodic use of cocaine “made [him] a weak person… irresponsible, unreliable”, prevented him from accomplishing goals and “finishing things”, and led to multiple divorces and incarcerations. He could not explain what drove him to return to cocaine after lengthy periods of abstinence, insisting only that so-called addictions experts had nothing to offer: “There’s no set pattern! [Someone might] stop [using] for a year, then go back to the same madness. So you never can tell what will happen.”

Echoing the model provided by diagnostic medicine, medical anthropology generally draws a distinction between the visible symptomatic dimension of illness and the underlying pathogenic processes of which it is a product (Engel 1977; Good 1994:8). The anthropological twist on the medical model has been to rearticulate this distinction between symptom and pathology—visible and invisible, manifest and latent—along the lines of the individual and the social. While arguably all medical anthropology follows this model in some form, calling attention to the social and cultural dimensions that mediate individual biological disease, this gesture is clearest in ‘critical’ medical anthropology’s efforts to theorize not just the “the cultural construction of symptoms and treatments”, but also “the social origins of disease” (Singer and Baer 2012:10). In this stronger formulation, individual symptoms are the direct product of oppressive social relations—manifest forms of ‘social suffering’ that result from latent processes of structural violence (Bourgois 1995; Farmer 2010; Kleinman et al. 1997; Biehl and Moran-Thomas 2009:275–276).

Critical medical anthropology, therefore—like perhaps all self-consciously ‘critical’ approaches—employs a hermeneutics of suspicion (Ricoeur 1970), in which symptoms are treated as appearances that must be demystified to reveal their true causes. Drawing on Lukacs, Michael Taussig argues that contemporary biomedicine *reifies* illness, mystifying the social relations that produce it. Discussing the case of a working-class woman suffering from polymyositis—a condition characterized by muscular degeneration—he notes how she understands her disease as the product of a lifetime of back-breaking domestic labor. While her lay explanation contradicts the biomedical explanations provided by her doctors*,* Taussig argues that it glimpses an essential truth: in drawing a connection “between polymyositis as muscle degeneration and her life-experience of oppression”, the disease comes to “stand as the arch-metaphor for that oppression” (1992:91), “pointing a finger of condemnation at a… personal experience of oppression” (Kleinman 1988:50–51, citing Taussig). Nancy Scheper-Hughes goes further in elaborating the ways that illnesses not only reify social relations but also communicate something through them. In her analysis of the folk ailment *nervos*, a nebulous illness encompassing nervous exhaustion and a host of debilitating physical symptoms experienced by the poor and malnourished in the Brazilian *favela*, Scheper-Hughes argues that while much of the symptomology of *nervos* is clearly the result of hunger, it is also “seething with meanings” (1993:173). Under conditions where political dissent is impossible, *nervos* becomes “an oblique form of protest” (213), in which symptoms, such as one man’s paralysis of the legs, come to communicate something essential about the oppression he experiences. Rather than being able to “face the world squarely, standing on one’s own two feet” (182), *nervos* communicates the “sinking, yielding, succumbing, giving up” (183) of those forced to “endure what is unendurable” (213). Finally, João Biehl (2005) demonstrates how illnesses, diagnoses, and pharmaceutical technologies are brought together in the management of kinship relations through his ethnography of Catarina, a woman suffering from an undiagnosed illness, living in a makeshift asylum for the destitute *abandonados* of Porto Alegre. As Catarina’s illness increasingly makes her a burden on her struggling migrant family, and as she refuses the subservient, gendered role that she is expected to play within it, she comes to be treated as mad, and psychiatry is used as means to pharmaceutically control her and eventually to remove her from the family and society. Biehl argues that Catarina’s illness, which is first diagnosed as schizophrenia and eventually determined to be an inherited neurological condition, must, in important ways, be understood as *relational.* It is a form of what he calls “social psychosis”, insofar as the ailment resides not in the individual’s physiology or psychology, but in “the actual struggles of the person to find her place in a changing reality vis-à-vis people who no longer care to make her words and actions meaningful” (Biehl 2005:18).

In these sophisticated readings, these authors suggest that illnesses must not simply be demystified, they must be *interpreted* as signifying practices that convey something about the social relations that produce them. Crucial, here, is the careful balance between demystification and interpretation—a balance that less sophisticated (or more polemical) versions of this argument often tip in favor of demystification. This snare is captured by Eve Sedgwick’s (2003) characterization of demystification as a form of ‘paranoid reading’. Sedgwick astutely diagnoses the “anticipatory” nature of overly hasty demystification as a suspicious posture that permits no surprises because it “requires that bad news be always already known” (130). Paranoid reading knows in advance what it will find, a demand that haunts many ‘critical’ approaches—arguably, mine included. My initial connection with Leon was formed precisely because of the alignment between our paranoid readings of his addiction. I was a researcher interested in the relationship between racism and drug addiction and he was a participant in a treatment program who was angry at the program’s resistance to critical, political analysis. We both started out knowing what we would find: that Leon’s addiction was the product of structural violence and racism. This meant that it took us a long time to perceive what else was at play. While the argument that I develop here is ultimately closer to the Jamesonian ‘symptomatic reading’ that Sedgwick critiques, what I caution against are modes of reading that place too much “faith in exposure” (138), presuming (like ‘vulgar’ Marxism or ‘wild’ psychoanalysis) that they can undo the false consciousness of illness through the revelation of mystified contents, without also engaging in the patient labor of interpretation.

It is within this double movement of demystification and interpretation that I situate the dreamwork as a model for analyzing how latent social contents are expressed in manifest symptomatic form. In proposing that we read an addiction as one might a dream, I take my bearings from critical reassessments of psychoanalysis that have sought to reimagine the relationship between surface and depth, arguing that truth is found not in the hidden depths of the subject, but on the surface of their discourse. While such critiques are often associated with forms of ‘surface reading’ emerging in the wake of Sedgwick’s intervention (e.g. Best and Marcus 2009; Felski 2015; c.f. Foucault 1996:56–57), they also have a long history within psychoanalysis (Copjec 1994; Dean 2002; Lacan 2006; Lear 2005; Leclaire 1998; Žižek 1989). Drawing on Lacan, this tradition cautions that “we must avoid simple metaphors of demasking, of throwing away the veils which are supposed to hide the naked reality” (Žižek 1989:28–29), for, as Joan Copjec puts it, “what is concealed may just as easily lie on the surface” (1994:170). What this critical psychoanalytic approach offers is a *formal* method of analyzing symptoms, one that seeks to reveal repressed contents not by unveiling but by analyzing the “surface mechanisms by which that content appears to have been hidden” (Dean 2002:33). The insights afforded by such an an approach became clear as I attempted to make sense of what Leon had to say, because, as I came to understand, the causes of his addiction did not require unveiling at all; they were, rather, all he talked about.

## Illness, Meaning and Relationality

Medical anthropology has long held that the problem of *meaning* lies at the center of the human experience of illness. As Kleinman and others have noted, contemporary biomedicine, despite its success in identifying the somatic causes of illness, is often unable to provide people with meaningful explanations for their suffering. Traditional healing practices, by comparison, address this question explicitly, offering explanatory frameworks for random, unfortunate events, as in Evans-Pritchard’s well-known analysis of Azande witchcraft (Evans-Pritchard 1972; Kleinman 1988:9; Good 1994:11–12; Taussig 1992:85). Leon devoted a great deal of energy to making sense of his addiction. While his 12-step treatment program focused on taking personal responsibility for one’s drug use and recovery, Leon saw political questions as essential to understanding addiction. Discussing the drug trade in the housing projects where he grew up, he asked provocatively: “From 1972 to 2000, millions and millions of dollars was made… what Black-owned stores are they? What [community] centers are put up?” Now that the projects had been largely demolished, he wondered:

Where are all the people… that was there? Where are they displaced to? What happened to ‘em? *They in the jails*. How did that happen? … Even though the crime rate fluctuates… the jail system is still eighty percent Blacks.

He further connected his ideas about mass incarceration to questions about urban renewal and gentrification:

Why is this neighborhood changing over a twenty-year period and the neighborhoods in Barrington [a wealthy suburb] haven’t changed over a hundred-year period? … Where are the old people—the guys that I used to see? Where are they at? They’re no more.

Leon’s political analysis drew together critiques of structural racism, mass incarceration, the drug war, and post-war urban renewal in terms that echo much contemporary critical scholarship (e.g. M. Alexander 2012; Davis 1997; Gilmore 2007; Wacquant 2000). At the same time, these progressive ideas were interwoven with conspiracy theory and reactionary ideas about feminism and homosexuality—an admixture recognizable from certain strands of Black Nationalism and the popular Afrocentrism of contemporary figures like Umar Johnson and Tariq Nasheed’s *Hidden Colors* series, which Leon recommended to me.^2^ Many of these ideas centered on narratives that connected the social problems of the inner city to a decline of patriarchal authority:

In the early 60s, the women depended on the man to take care of the family. Then, when public aid was at its height … the woman is independent, and they don’t have to depend on the man, then she can just about do anything she wanted to do… and if they can take birth control, then they can have sex with whoever they wanted to and not have to have the man… Morale breaks down in the family, which spreads to other families, and eventually the neighborhood… too many components were brought in: Birth control. Drugs. No morale in the family at all. No *leadership*… It’s a domino effect.

Leaving to one side the critiques that one could make of this analysis (which recalls Daniel Patrick Moynihan’s (1965) notorious characterization of ‘matriarchal’ African American families as a “tangle of pathology”), these ideas played an important role in helping Leon make sense of his addiction by providing a framework to explain the devastating changes that took place in African American communities in the wake of the civil rights era.^3^

They also informed his critiques of the residential 12-step treatment program where I met him, which he attended as a requirement of his parole. Rather than focusing solely on an individual’s drug use, he argued, treatment needed to consider broader political questions, such as “How did the drugs get *into* the neighborhood?” and “Why does the drugs have such a great effect on the Afro-American than they do on the Anglo-Saxon?” Discussing these political questions, he believed, “would put a different mind-set of the people that come here”. Nor were his critiques limited to politics; as if channeling the arguments of many critical scholars of addiction (e.g. Dodes and Dodes 2014; Peele 1985; Szalavitz 2016), Leon rejected the insistence in 12-step treatment that drug users should permanently conceive of themselves as addicted: “Their philosophy… says… ‘I’m an addict’ … You come here to be healed [but] you continue to say you sick! At what point in time are you healed?” Refusing to take on such a stigmatizing identity, he chose to see himself, evocatively, as “a new creature”.

Like a good medical anthropologist, Leon had a well-developed critique of the reified account of addiction found in conventional approaches to treatment, and this critique provided him with a meaningful framework through which to understand his addiction. He had, in other words, already demystified the social causes of his illness. And yet, these explanations failed to provide him with what he needed most from them. Not only did his relationship to drugs remain unchanged (admittedly, a tall order for demystification alone), but they also failed him at the level of *meaning*. Leon’s socio-political explanations faltered when he considered the problem of why he was addicted to drugs while other people in his life, specifically his successful brother, were not. It was this question that marked the limit of his narrative’s explanatory power. If racism and politics were determinant, he reasoned, then both he and his brother should have suffered the same fate; yet while his life had been turned upside down by drugs, his brother had thrived. This apparent contradiction profoundly disturbed his attempts to answer the question ‘Why me?’ that Arthur Kleinman argues lies at the heart of illness. Kleinman calls this the “problem of bafflement” (1988:29): the fundamental question for the sick person that contemporary biomedicine fails to address. But while Kleinman poses this question in existential terms, as a problem of finding meaning in suffering, Leon reframes the question in relational terms, asking ‘Why me *and not my brother*?’.

The counterfactual presented by his brother’s success caused Leon to speculate about what else was potentially significant to his addiction—most notably, his family. Leon grew up as the eldest male child in the household; along with his mother and father, he had two younger brothers as well as two sisters, one older and one younger. He described his mother as “a hardworking worker” who raised the children while employed, first in factories and then, after further education, as a dental hygienist. His father was a more ambivalent figure. He worked as a laborer and sometimes a welder, a profession that Leon followed him into. He also gambled and drank and spent significant periods away from the family, habits that Leon would repeat, in his own way. When Leon was ten years old, his parents divorced and his father moved out. Shortly afterwards, his mother sent him to live with relatives in small-town Ohio, in part due to concerns about the increasing gang culture in the neighborhood. He returned two years later, homesick, to find his mother with a new partner. Seeing “another man standing in the house” rankled the teenaged Leon and he soon moved out, going to live with his father and becoming “rebellious” in his teenage years, skipping school and smoking marijuana.

Leon’s account of his family history is suggestive of a complex series of identifications with his mother and father that appear to bear directly on his later drug use. In this narrative, he connects his drug use to his father’s drinking, while his successful brother identified with their mother:

My mother was a hardworking worker and… she instilled that into my brother… He helped her out, he worked. I went the other way. I went off with my family, and then I got into drugs… My father drank… I followed his pattern. My brother followed my mother’s pattern.

This account of Leon’s childhood allows us to consider a different set of latent contents that make up his illness narrative. Here, his addiction is the manifest expression not simply of structural racism but also of unresolved childhood issues related to his parents’ divorce, his father’s drinking, his rivalry with his brother and their parallel identifications with their parents, which depict a binary choice between the industrious mother and the beloved yet unreliable father. Leon knew that his family and his experience of structural racism both figured in his addiction. The point at which these two explanatory frameworks—the political and the familial—intersected, however, created great difficulties for him, opening a troubling aporia. He returned to it repeatedly in our interviews, often sliding from one explanation to the other before perceiving an impasse and breaking off his narrative:

Why did I use drugs? Would I have used drugs if they wasn’t in the neighborhood? Probably not. [Long pause] But can I really blame the drugs… being in the neighborhood? Can I? I wonder. I wonder. I wonder. If they would’ve been in another neighborhood, would I have went and searched for ‘em?

And then, during a later interview:

All of it leads to the drugs… I’m a product of my misfortune. ‘Cause the drugs was in the neighborhood. *I used and my brother didn’t*—so I just don’t put the blame on, on just ‘cause the drugs was there. I put the blame on myself… But I’m just saying— … we can’t say what would have happened to me if it—if the drugs wasn’t in the neighborhood. Just can’t say that.

Leon, then, was confident in his political analysis, but troubled by the question of how much of his own situation could be explained by it. One simple answer could be that both explanations are true: behind the manifest content of Leon’s symptom lie two distinct latent contents—structural racism and family history—that must be demystified. We could also observe that brute social facts such as racism affect people in different ways. As Paul Rabinow argues, in a discussion of colonialism:

What at first seem to be the broadest and richest concepts, capable of organizing and clarifying the most material turn out to be the most impoverished. The passage from broad assertions […] to individual cases must be mediated by particular determinations, because otherwise there is no way to differentiate one village… from another (Rabinow 2007, 121–22).

Differentiating one village—or in Leon’s case, one brother—from another is an essential part of the analysis of symptoms. Arthur Kleinman (1988:240) argues that illness narratives are often “incoherent”, so we should not be surprised if Leon’s narrative is too. But rather than attempting to explain away the “incoherence” in Leon’s illness narrative through demystification, I want to argue that this incoherence is itself worthy of our attention. Leon’s competing illness narratives have an internal logic, and by analyzing it, we can understand his illness more completely. This logic is captured by Freud’s idea of the dreamwork.

## The Dreamwork

Freud’s method of interpreting dreams (and symptoms) has long been treated as a process of demystification, in which repressed, unconscious contents are unveiled by a masterful interpreter (Ricoeur 1970; Dean 2002; Lear 2005; Žižek 1989). Freud famously argues that what we remember of our dreams, the ‘manifest content’, is a distorted re-telling of the latent ‘dream thoughts’ and unconscious wishes that are its true source. These latent thoughts are subject to condensation, displacement and other operations of the ‘dreamwork’, which disguise them, allowing us to continue our sleep without confronting the often distressing nature of our desires. Uncovering the latent content of dreams, however, must not be treated as the end point of analysis, as Freud states in a crucial footnote to the *Interpretation of Dreams:*

Now that analysts at least have become reconciled to replacing the manifest dream by the meaning revealed by interpretation, many of them have become guilty of falling into another confusion… They seek to find the essence of dreams in their latent content and in so doing they overlook the distinction between the latent dream thoughts and the dream-work… It is the dream-work which creates that form, and it alone is the essence of dreaming. (Freud 1900, 506–7)

Here, Freud argues for a more formal analysis of dreams than is implied by the content-centered metaphor of unveiling. Rather than concluding our analysis when we arrive at the latent thoughts behind the manifest content, Freud insists we attend to the mechanisms by which one is transformed into the other. As Slavoj Žižek puts it: “The point is to avoid the properly fetishistic fascination of the ‘content’ supposedly hidden behind the form: the ‘secret’ to be unveiled through analysis is not the content hidden by the form… but, on the contrary, *the ‘secret’ of this form itself*” (1989:11﻿). Freud’s insistence that the dreamwork constitutes the essence of dreaming provides another way to consider the problem of demystification: interpretation cannot consist simply of revealing the latent content concealed by the manifest; rather, it must concern itself with the formal mechanisms of their translation. Key to this argument is the often-ignored fact that latent contents are rarely hidden in the ways we imagine. As Jonathan Lear observes, in nearly all of Freud’s best-known examples, the latent thoughts that give rise to dreams are not repressed desires but everyday thoughts. In Freud’s dream of the botanical monograph (1900:169–176), for example—a dream that connected Freud’s present-day professional ambitions to Oedipal conflicts from his childhood—Lear notes that the “blistering childhood memory” of being chastised by his father that makes up a central part of its latent content “is not itself repressed… What is missing… is not any particular memory, but a sense of how it all fits together” (2005:97).

Like Freud’s dream, the latent contents of Leon’s addiction were not in any way repressed. Leon was very much aware that structural racism and his family had both played a role in his drug use; what eluded him was how these two narratives might fit together. Much as medical anthropology has aimed at reconnecting the social relations that lie behind illness, I argue that what is required here is to re-establish the connections between narratives. If ‘repression’ means anything in this context, it consists of the severing of these connections, not the repression of the contents themselves (Fink 2017:55–63; Freud 1909:196). In order to understand Leon’s addiction, we need to examine the gaps, omissions, and elisions between his narratives, to see how they are brought together in the dreamwork of his symptom.

## The Dreamwork of Addiction

Leon presents us with two narratives. In one, his drug use is the product of structural racism and the collapse of patriarchal authority. In the other, it is the result of an identification with his father in contrast to his brother’s identification with their mother. These narratives are not wholly distinct, of course. Many defining features of Leon’s family life bear the stamp of structural racism, including his experiences growing up in Chicago’s underfunded public housing system, the increasing gang activity that led to his being sent away to live with relatives, and, most significantly, his relationship to his father. While Leon and I did not discuss his father in enough depth for me to assess this, it is entirely plausible that his father’s absences, drinking, gambling, and somewhat precarious employment were themselves a product of, or at least related to, his experience of structural racism. Indeed, it is difficult to imagine otherwise.^4^ Leon’s family narrative, therefore, cannot be neatly separated from his political narrative.

Nevertheless, as I have noted, Leon himself perceived a contradiction between these two explanatory frameworks: he knew that the conclusions he had drawn did not all square with one another. Rather than seeing these narratives as contradictory, the dreamwork suggests another way to read them: as condensations and displacements of one another. Displacement describes the unconscious process by which the affects associated with one idea becomes attached to other ideas in a chain of associations, while condensation describes how different associative chains are superimposed on one another and come to be represented by a single overdetermined link or idea (Freud 1900). Viewed in this way, it becomes possible to read aspects of Leon’s political analysis as another way of telling the story of his family. For example, the historical narrative he presents of the collapse of patriarchal structures and the empowerment of women precisely mirrors his childhood, in which his father’s failure to occupy the position of authority meant that it was his mother who took on this role. Leon responds to this experience by developing a political analysis that repeats, in distorted form, the experiences of his childhood: ‘Things go wrong when women become dominant’. If we read this analysis literally—that is, on its surface—we are presented with a description of his experience of his parents’ separation: his father left the house, his mother became the primary breadwinner, and she began a new intimate relationship—a change that he, as the oldest male child, may have perceived as the usurpation of his nascent patriarchal position within the household (“another man standing in the house”). The powerful affects that accompanied these politics—evident in the passion with which he spoke of them—could likewise be understood, at least in part, as a displacement of the emotions occasioned by these early experiences. Following Kleinman, these elements of Leon’s politics may be read as a retroactive attempt to impose meaning on that experience (Kleinman 1988:240; see too Frosh 2007:638).

To be clear, I am not arguing that Leon’s politics are an example of false consciousness or that his politics are ‘really’ about his family—not least because I agree with many aspects of his analysis (and it is notable, perhaps, that it is the misogynistic, and not anti-racist, aspects of Leon’s politics that are most suggestive of unconscious repetition). Rather, I am arguing that viewing these competing narratives through the lens of the dreamwork allows us to see multiple forms of connection, rather than contradiction, between them. Of some of these connections, Leon is well aware, whereas others have been severed and are no longer accessible to consciousness—for reasons that are worth exploring. One possibility is that this condensation and displacement occurred because, while Leon appeared unconflicted about his politics, he was, like many of us, more ambivalent about his family. The translation of these familial conflicts into the sphere of politics might have made it possible for him to manage the anxiety they provoked, much as Obeyesekere argues that the “work of culture” is the “process whereby painful motives and affects… are transformed into publicly accepted sets of meanings and symbols” (1985:147; Delvecchio Good and Good 1988:58). In similar terms, Leon transforms the distressing experiences of his childhood into a political analysis.

If the dreamwork helps us to understand something about the content of Leon’s illness narrative, more striking still is what it shows us about its form. The formal properties of Leon’s addiction are, in many ways, its most distinctive feature: in particular, the extended periods—often years—of abstinence between binges. While alternating patterns of use and non-use are not uncommon in many (in particular, stimulant) addictions, Leon was nevertheless notable for the extended periodicity of his cycles. The conventional way of reading such a symptom is to see a man addicted to cocaine who manages for long periods of time to abstain from using it. This reading assumes that it is the *use* of the drug that is most important. Reading the symptom on its surface, however, might suggest that both states are significant, with abstinence being as central to the addiction as the binge. Freud (1909) argues that neurotic symptoms are the product of a conflict between opposing forces, one of which generally remains conscious, while the other is repressed. This conflict may be expressed in a ‘compromise formation’ in which the two opposing forces appear simultaneously, in distorted form; or, they may take the form of a symptom that *alternates* between two states, as in Freud’s case of the ‘Rat Man’, whose ambivalence towards his loved ones resulted in alternating acts of conscious altruism and unconscious aggression (1909:192; Fink 2017:55–63). Leon’s use and non-use, binge and abstinence, can be read as just such an “alternating symptom” (Fink 2017:202, 212), in which both states are meaningful. I suggest that this aspect of his symptom can be understood as an oscillation between the different meanings that Leon associated with his drug use: between hard work and hedonism; between respectable, loving relationships and transgressive, selfish pleasures; between his avowed politics and their opposite—oppositions that echo Freudian dyads of conscious and unconscious, ego and id, reality principle and pleasure principle. This oscillation can also be understood as a question of identification, in which periods of abstinence represent an identification with his hardworking mother, while his binges are an identification with his unreliable father. When one impulse or identification predominated, Leon worked, pursued his education, maintained his relationship with his wife and children, and lived up to his ideals; when the second was ascendant, he binged on drugs, stole, neglected his relationships, and abandoned his life projects. Leon’s description of an ‘addict’ evoked this, describing the qualities of the addict in direct contrast to his ideal of patriarchal reliability: “When you’re a addict, you’re not dependable… A addict steal, a addict lie. A addict is not responsible.”

These competing identifications are all the more significant because they confronted Leon with what appeared to be one of his most fundamental quandaries: that it was his mother rather than his father who possessed the qualities he saw as essential to being a man. To be a man demanded that he identify with his mother—a prospect that troubled him, and against which his rigid gender politics may be read as a defense. Nancy Scheper-Hughes argues that the symptom is constructed on what she calls the “generative metaphor”, around which “radiate a set of core oppositions” (1993:187–188). For Leon, this may well be the question ‘What does it mean to be a man?’—a conflict that gives rise to the binaries of female/male, responsibility/unreliability, strength/weakness, work/pleasure that organize his addiction. The crucial subtext behind Leon’s version of this already-difficult question is an experience familiar to many children of divorce: choosing between his mother and his father. Rather than answer it, Leon’s solution is his symptom, a non-choice that alternates between identifications, between responsibility and unreliability, between embracing and rejecting the role to which he aspires—between doing the right thing and fucking it all up. His symptom may thus be understood as a protest against the choice he feels he is forced to make, in the form of a refusal to make any choice at all—a refusal, as he put it, to “finish things”. His addiction is a rebellion against the Other’s demands and an indictment of the impossible position he has been forced to occupy (Fink 2017:27, 131, 205).

Read in this way, we can understand the relationship between Leon’s familial and political narratives as one in which his illness is the obverse of his politics. In his politics, Leon consciously protests something that, in his symptom, he unconsciously repeats. Describing the world that Leon grew up in, Loïc Wacquant (2000) argues that mass incarceration marked a shift in the state’s relationship to African Americans. As deindustrialization rendered a large part of the working class surplus to the needs of capital, mass incarceration transformed a racialized population that had formerly been *contained* in the ghetto and *exploited* by capital into one that was now *confined* to the prison and *excluded* from society (see too Davis 1997). This collective, inter-generational trauma is, at one level, what Leon arguably wrestles with in his politics and his addiction. To resist is one option, of course, and Leon does indeed resist through his politics. But the unconscious obverse of resistance is to embrace one’s status as surplus, as expendable, as unreliable. This is something that Leon might be understood to do through his symptom. As Freud (1909:180) often notes, a fear is always intertwined with a wish, and the same might be said of protest and its opposites.

## Conclusion: The Right to an Unconscious

The dreamwork offers a mode of reading capable of bringing together a range of ideas about the meanings of symptoms, from the humanistic assertion that the search for meaning is a central concern of illness, to critical efforts to uncover the latent social relations behind symptoms. To these hermeneutic endeavors, the dreamwork offers a twist on the question of meaning. Paraphrasing Lacan, Owen Hewitson argues that the dream does not so much “*have* a meaning” as “it is the production of a meaning” (2020:n.p.). Translated into the language of surface and depth, this means that rather than concealing a hidden meaning that must be unveiled, the dream articulates a meaning in the form of its enunciation. In similar terms, we can understand symptoms not simply as the products of latent social forces but also as sites for the production of meanings. One of the most striking features of Freud’s formal analysis of dreams is his insistence that what often appear as judgments *about* their content should instead be understood as part of the latent content itself (1900:445–452). For example, when analyzing a dream that one analysand insisted was “unclear”, Freud observes that “the lack of clarity shown by the dream was part of the material which instigated the dream”, for it pointed towards the dreamer’s own uncertainties about the latent issues that made up the dream. This leads Freud to the conclusion that “*the form of a dream* […] *is used with quite surprising frequency for representing its concealed subject matter*” (1900:332 emphasis in original). It is this formal reading that, to me, sheds the most light on the seemingly unresolvable contradiction between family and politics that Leon perceived in his symptom: it should be understood not as a judgment about the relationship between two latent contents, but as part of the latent content itself. As I have argued, these two narratives are not unresolvable: both narratives can be, and indeed are, true. Rather, the apparent contradiction that Leon encounters in his illness narratives is a stand-in for the real intractability of his situation: as an African American man exiting the prison system, as a person struggling with addiction, and as the particular person that he is within his family. It is as if the incommensurability of his narratives provides him with a way to declare: ‘impossible’, irresolvable’—a message to the Other that he communicates through his symptom itself.

What can we say about the truth value of such an interpretation? In a psychoanalytic context, Bruce Fink argues that “an interpretation is only as good as the progress it leads to; and it proves to be completely useless if, no matter how complete and seemingly exhaustive, it brings no change or fresh food for thought” to the analysand (2017:109–110). As Leon and I were engaged in interviews rather than analysis, we cannot look to this methodological standard to guide us; moreover, for practical reasons, I am no longer able to share these interpretations with Leon and hear his thoughts. Translating Fink’s argument into the context of social science, perhaps the value of an interpretation lies simply in what new thinking it makes possible. As Jonathan Lear puts it, while “we are not in a position to know for sure whether [my] interpretation is correct; the interesting point is that it could be” (2005:101).

In closing, I suggest that alongside the accuracy and truth of an interpretation, there is an equally important place for an idea common to both medical anthropology and psychoanalysis: what Kleinman (1988) calls ‘incoherence’ in illness narratives, and Lacanian psychoanalysis addresses in its critique of *understanding* (Fink 2014; cf. Scheper-Hughes 1993:170). As Bruce Fink argues, “Understanding… virtually always involves jumping to conclusions about things we do not yet fully understand… to reduce something [unknown] to what we already know” (2014:11). This is why Tim Dean asserts that ‘rather than making sense of trauma, psychoanalytic interpretation draws attention to its resistance to sense’ (Dean 2002:34; see too Lapping and Glynos 2019). Leon’s case demonstrates why researchers should be wary of reaching too quickly for understanding at the expense of *non-meaning.* Leon’s case was complex and I struggled to understand it for years afterward, despite the many explanations that he and I circled around. This is perhaps part of the problem: people in treatment for drug addictions are rarely lacking in ‘understanding’ about their symptoms. Meaning is foisted upon them at every turn, in the form of ideas about addictive personalities, hijacked dopamine pathways, spiritual maladies, or indeed, structural violence. Such meanings are not to be discounted, of course. As Stephen Frosh argues:

Rebuilding narrative coherence is an estimable goal for those who find themselves on the margins of hegemonic discourses... Just as individuals benefit in the psychotherapeutic domain from being able to speak their stories and have them reflected back in a way that enables them to be owned, so in the political domain it is precisely through the coherent articulation of subjugated narratives that oppressed groups become empowered.” (2007, 637; see too Saville Young and Frosh 2010).

Such efforts at meaning-making not only provide the necessary foundation for political action, but they also lie at the very heart of what it means to be human. The problem is that these meanings are often *paranoid*, in Sedgwick’s sense—reaching for conclusions where they could open further questions. As Freud discovered early in the development of psychoanalysis, providing a patient with the ‘meaning’ of their symptom rarely leads to its dissolution; all too often, a seemingly exhaustive interpretation provokes only resistance, or leads to the appearance of new symptoms in place of the old. Leon protested the meanings offered to him by 12-step philosophy, but only so that he could substitute meanings of his own—meanings that I was all too willing to agree with initially. As a researcher, I fell into the trap that Lacan identifies when he notes that analysts “always understand too much” (1988:103), asserting that “the less you understand, the better you listen” (141).

As I have argued, a crucial task for critical health research is to make space for the unconscious lives of marginalized people. This right to an unconscious goes hand in hand with the right to incoherence and complexity—it is the entitlement to be more than one’s oppression. Leon and I both understood his problem in advance, which is why we failed to fully hear it: racism and structural violence *were* central to his addiction, but these forces articulated themselves in ways that were particular to his life history. His symptom tied these registers together, linking the personal to the political in a form that was as singular to him as it was expressive of broader structures. It is to these particularities and opacities that Paul Rabinow refers at the end of *Reflections on Fieldwork in Morocco*, when he warns other would-be critical researchers that “what the ‘facts’ demonstrated was far from obvious… [and] what seemed… to ‘speak for itself’ proved to be the most in need of interpretation”. Interpreting symptoms in light of structural causes requires us to trace Rabinow’s “*particular* mediations” of social forces to ensure that they are not reduced to “sterile truisms” (2007:124). Leon was indeed entitled to be angry at what had happened to him. In asserting his right to an unconscious, I am arguing that he deserved even more space for that anger to be articulated: not just in righteous protest, but in all the complex and contradictory ways that traumatized people survive, make sense of their experience, and find joy.

## Footnotes

1 A pseudonym. Some identifying details have been altered to ensure anonymity.

2 On Black nationalism and gender see, for example: Davis (1988:181–182), Hooks (2015), Mutua (2006), Young (2016). A more generous reading of why some Black men adopt sexist frames of analysis can be found in Orisanmi Burton (2021) and in particular his discussion of this work at: https://millennialsarekillingcapitalism.libsyn.com/a-profound-tenderness-orisanmi-burton-on-black-masculine-care-work-within-zones-of-war. My thanks to Ted Rutland for this reference.

3 Among the many Black feminist critiques of the Moynihan report, see, for example: Davis (1983:4–15), King (2018), Spillers (1987).

4 For critical reflections on the figure of the ‘absent father’ in African American communities, see: Alexander (2012:178), Dyson (2008), King (2018).
